# P-248. Novel and Potential Biomarkers in prediction and Prognosis of Cryptococcal-IRIS: A systematic review

**DOI:** 10.1093/ofid/ofaf695.470

**Published:** 2026-01-11

**Authors:** Devrakshita Mishra, Nafisa Alam, Sameer Kumar Majety

**Affiliations:** School of Medicine, Xiamen University., Delhi, Delhi, India; School of Medicine, Xiamen University., Delhi, Delhi, India; School of Medicine, Xiamen University., Delhi, Delhi, India

## Abstract

**Background:**

Cryptococcal immune reconstitution inflammatory syndrome (C-IRIS) remains a life-threatening complication in HIV-infected individuals initiating antiretroviral therapy (ART). We conducted a systematic review to identify immune and molecular biomarkers predictive of C-IRIS.

**Image 1:**
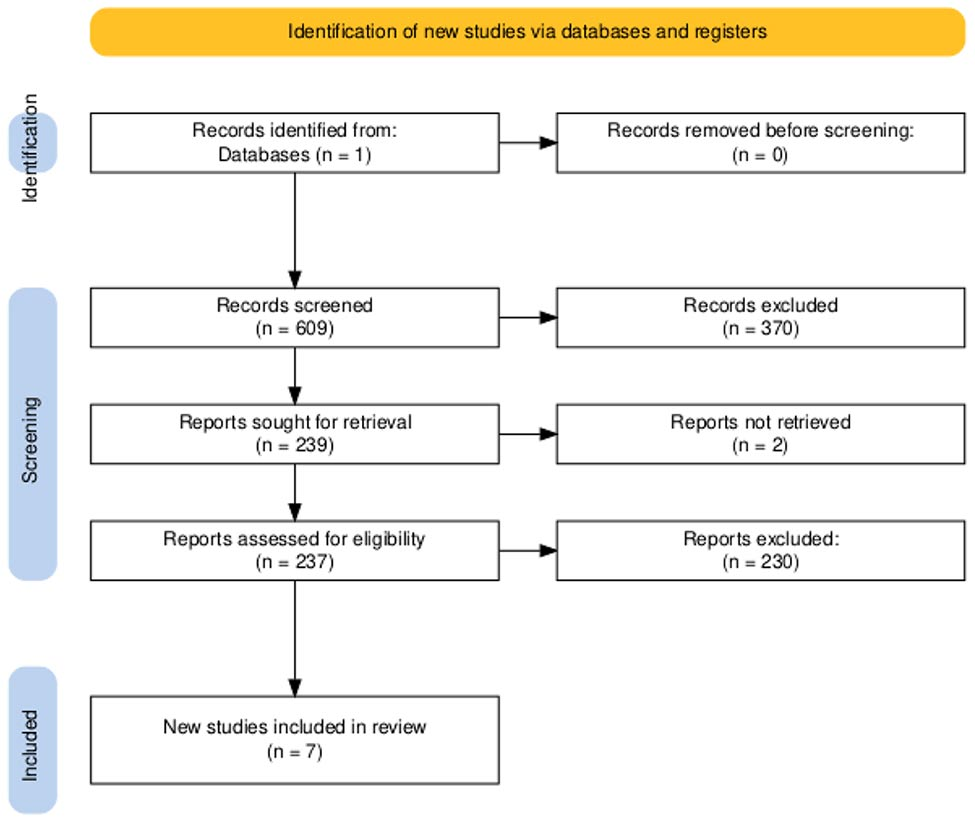
PRISMA flow diagram illustrating the study selection process for the systematic review.

A total of 609 records were identified through PubMed. After title and abstract screening, 238 full-text articles were reviewed. Of these, 19 studies focused on cryptococcal IRIS, and 7 met inclusion criteria for evaluating predictive biomarkers.

**Table 1:**
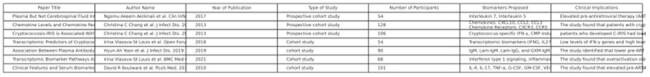
Summary of studies evaluating biomarkers associated with cryptococcal IRIS in HIV-positive individuals.

The table includes seven studies (2010–2021) assessing clinical, immunologic, and transcriptomic biomarkers predictive of IRIS onset or severity. Key biomarkers include cytokines (IL-4, IL-5, IL-7, IL-17), chemokines (CXCL10, CCL2, CCL3), antibody levels, and transcriptomic markers (e.g., IFNG, IL27). Most studies were cohort designs with sample sizes ranging from 54 to 128 participants. Reported clinical implications highlight associations between pre-ART immune dysregulation and subsequent IRIS development.

**Methods:**

Following PRISMA guidelines, we searched PubMed for articles published between January 2010 and January 2025 using the terms: “(((IRIS) OR (Immune Reconstitution Inflammatory Syndrome)) AND ((HIV) OR (Human Immunodeficiency Virus))) NOT (Case Report) NOT (Review).” Of 609 records screened, 238 full-text articles were reviewed for eligibility. Nineteen studies addressed cryptococcal IRIS; seven met inclusion criteria by evaluating biomarkers associated with C-IRIS development (total N=601).

**Results:**

Elevated plasma IL-5 (HR=5.76) and IL-7 (HR=9.30) levels pre-ART were strong predictors. CSF markers included CCL2/CXCL10 chemokine ratios (P=0.02) and CXCR3+CCR5+ CD8+ T-cell trafficking. IRIS patients showed impaired pre-ART IFN-γ responses (P=0.0437) and reduced cryptococcal-specific IgM/IgG (P< 0.005). Transcriptomic profiling revealed pre-IRIS downregulation of IFNG, IL27, KLRB1 and upregulation of AIM2, BEX1, and C1QB. Serum IL-4, IL-17, and CRP >32 mg/L further stratified risk. Fatal cases demonstrated activation of inflammasome and TLR pathways. No biomarker achieved sensitivity or specificity above 80%. All studies were observational with heterogeneous methods.

**Conclusion:**

Despite emerging multi-omic signatures, no single biomarker reliably predicts C-IRIS. Integration of transcriptomic, cytokine, and antibody data may enhance future risk models. Prospective validation using standardized protocols is needed.

**Disclosures:**

All Authors: No reported disclosures

